# Comprehensive organic profiling of biological particles derived from blood

**DOI:** 10.1038/s41598-018-29573-6

**Published:** 2018-07-27

**Authors:** Cheng-Yeu Wu, Jan Martel, John D. Young

**Affiliations:** 1 Laboratory of Nanomaterials, Chang Gung University, Taoyuan, Taiwan; 2 Center for Molecular and Clinical Immunology, Chang Gung University, Taoyuan, Taiwan; 3 Research Center of Bacterial Pathogenesis, Chang Gung University, Taoyuan, Taiwan; 4 Chang Gung Immunology Consortium, Linkou Chang Gung Memorial Hospital, Taoyuan, Taiwan; 5 Biochemical Engineering Research Center, Ming Chi University of Technology, New Taipei City, Taiwan

## Abstract

Mineral nanoparticles form in physiological and pathological processes occurring in the human body. The calcium phosphate mineral phase of the particles has affinity for proteins and lipids, but the complete profiling of the organic molecules that bind to the particles has not been described in detail. We report here a comprehensive analysis of organic components found in mineralo-organic particles derived from body fluids. Based on biological staining, fluorescent tagging, proteomics and metabolomics, our results indicate that the mineral particles bind to proteins, amino acids, carbohydrates, polysaccharides, phospholipids, fatty acids, DNA and low molecular weight metabolites. These results can be used to study the formation and effects of mineralo-organic particles in biological fluids.

## Introduction

Mineral nanoparticles (NPs) have been shown to form in human biological fluids when the concentration of calcium, carbonate and phosphate ions exceeds saturation^1–19^. These mineral particles represent the first mineral that forms in bones and teeth, as well as in ectopic calcifications and kidney stones^13^. The particles have been detected in the body fluids and tissues of patients suffering from various conditions, including atherosclerosis, chronic kidney disease and type 2 diabetes^5,13^. As such, the particles represent precursors of physiological and pathological mineralization processes that occur in the human body.

Recent studies indicate that the mineral particles participate in other physiological roles *in vivo*. For instance, the particles form in the lumen of human intestines where they bind to dietary antigens and bacteria-derived molecules such as peptidoglycan; the particles in turn present these molecules to macrophages and dendritic cells of the intestinal mucosa to induce immunological tolerance against food and the gut microbiota^20^. Similar mineral particles called calciprotein particles (CPPs) have also been detected in ruptured fetal membranes from idiopathic preterm birth, suggesting that the particles may also affect human fertility and fetal development^21^.

We suggested earlier that mineral particles forming in the human body may influence physiological processes by binding to various organic molecules in biological fluids^13^. Accordingly, proteins bind to the particles and form a “protein corona”^7^, possibly influencing the levels of these proteins in biological fluids. Identification of the molecules that bind to the mineral particles is needed to assess the effects of the particles’ organic components *in vivo*. Based on various techniques including biological staining, fluorescent tagging, electrophoresis, chromatography, proteomics and metabolomics, we present here a comprehensive analysis of the organic corona of mineral particles formed in bovine and human serum.

## Methods

### Preparation of particles

The use of human body fluids was approved by the Institutional Review Board of Linkou Chang Gung Memorial Hospital. Written informed consent was obtained from the healthy volunteers who provided the samples. The experiments were performed in accordance with the relevant guidelines and regulations. Human blood was withdrawn and serum was prepared as described before^2^. Particles were prepared by adding 3 mM of CaCl_2_ and Na_2_HPO_4_ each to Dulbecco’s modified Eagle’s medium (DMEM; Gibco; final volume of 1 ml) containing or not 10% (v/v) fetal bovine serum (FBS; Biological Industries) or human serum (HS). Following incubation in cell culture conditions overnight, the particles were centrifuged at 16,000 × g for 10 min and washed with 20 mM HEPES buffer containing 1 mM CaCl_2_, 2 mM Na_2_HPO_4_, 150 mM NaCl (pH 7.4). Particles were resuspended in 0.5 ml of double-distilled water or 50 μl of 0.5 mM EDTA.

### Electron microscopy and spectroscopy analyses

Electron microscopy and spectroscopy analyses were performed as before^2^. Briefly, washed samples were deposited on Formvar carbon-coated grids and excess water was removed. Dried samples were observed under a field-emission S-5000 scanning electron microscope (Hitachi). Energy-dispersive X-ray spectroscopy (EDX) was performed using an EMAX Energy EX-400 EDX device and the software provided by the manufacturer (Horiba). For Fourier-transform infrared (FTIR) spectroscopy, dried samples were mixed 1:100 (w/w) with KBr and analyzed using a Nicolet 5700 FTIR spectrometer (Thermo Fisher Scientific). For Raman spectroscopy, dried samples were examined using the inVia Raman confocal microscope (Renishaw) equiped with a 50 × objective and a charge-coupled device detector. For X-ray diffraction analysis, dried samples were studied using the D5005 X-ray diffractometer (Bruker) and the obtained spectra were compared with the Joint Committee on Powder Diffraction and Standards.

### Coomassie blue staining

Particles (50 μl) were mixed with 50 μl of Coomassie blue staining solution (0.1% w/v Coomassie Brilliant Blue R-250, Sigma; 50% v/v methanol and 10% v/v glacial acetic acid), followed by gentle mixing using the Intelli-Mixer RM2 (Elmi) for 30 min at room temperature. The solution was centrifuged at 16,000 × g for 10 min, and the pellet was resuspended in 500 μl of destaining solution (50% v/v methanol, 10% v/v acetic acid). The solution was mixed for 30 min as above. Destained particles were collected by centrifugation and washed three times with water. Particles were resuspended in 20 μl of water, and 10 μl of the solution was deposited onto a glass slide, covered with a cover slip, and observed under a BX-51 optical microscope (Olympus).

### Oil red O staining

A stock solution of oil red O stain was prepared by dissolving 0.5 g of oil red O dye (Merck Millipore) in 100 ml of isopropanol in a warm water bath. The staining solution was prepared by mixing 30 ml of the stock solution with 20 ml of water. The solution was allowed to stand at room temperature for 10 min, prior to filtration into a Coplin jar. The oil red O staining solution (50 μl) was mixed with particles (50 μl) and incubated for 30 min at room temperature. After staining, particles were collected by centrifugation and washed three times with water. Particles were resuspended in 20 μl of double-distilled water and observed using optical microscopy.

### Alcian blue staining

The staining solution was prepared by dissolving 1 g of alcian blue 8GX (A5268, Sigma) in 100 ml of 3% (v/v) glacial acetic acid. The pH was adjusted to 2.5 and the solution was filtered into a Coplin jar. The alcian blue staining solution (50 μl) was mixed with 50 μl of particles, and the solution was incubated with gentle agitation for 30 minutes at room temperature. Particles were collected by centrifugation and washed three times with water, prior to observation under optical microscopy.

### SYBR gold staining

The SYBR gold nucleic acid staining solution (S11494, Invitrogen) was diluted in water (2.5 μl of the 10,000 × SYBR solution into 1 ml of water). An aliquot of the solution (30 μl) was mixed with particles (50 μl) and incubated with gentle agitation for 30 min at room temperature in the dark. After staining, the particles were colleted by centrifugation and washed three times with water. The particles were resuspended in water (20 μl). Microscopic observations were conducted with an epi-fluorescence microscope (AX70, Olympus) using a blue broad-band filter for excitation (488 nm).

### Binding assays

Bovine serum albumin (BSA; 10 mg; Sigma) was dissolved in 1 ml of water and stored at −20 °C before use. Phosphatidylethanolamine (25 mg; Sigma) was dissolved in 2 ml of chloroform; the solution was dried in a vacuum centrifuge, and 10 ml of water was added, prior to mixing (final concentration of 5 mg/ml). Xanthan gum (0.5 mg; Sigma) was dissolved into 1 ml of water. Salmon sperm DNA (0.3 g; Sigma) was dissolved into 30 ml of water with gentle mixing for 4 hrs at room temperature. Residues were dispersed using a syringe with a 18 G needle. Water was added to obtain a final concentration of 1 mg/ml of DNA.

Particles were also prepared by adding 3 mM of CaCl_2_ and Na_2_HPO_4_ each to DMEM (1 ml, final volume) containing 0.1 to 100 μg of BSA, as well as FBS (10%), phosphatidylethanolamine (25–250 μg), xanthan gum (0.04–20 μg), or salmon sperm DNA (1–30 μg). Following incubation in cell culture conditions for 30 min, the particles were pelleted and washed with 20 mM HEPES buffer (1 ml), prior to resuspension in 50 μl of 50 mM EDTA.

### Sodium dodecyl sulphate-polyacrylamide gel electrophoresis

Washed particles were resuspended in EDTA and mixed with 4 × sample denaturing buffer (15 μl), prior to heating at 95 °C for 10 min. Samples (10 μl) were submitted to 10% sodium dodecyl sulfate-polyacrylamide gel electrophoresis (SDS-PAGE). Protein staining was done with Coomassie blue as described earlier^2^.

### Thin-layer chromatography

Particles prepared as above using DMEM (1 ml) containing 10% FBS or 25–250 μg of phosphatidylethanolamine were washed and resuspended in EDTA. The solution (20 μl) was loaded onto a 10 cm × 10 cm pre-coated thin-layer chromatography sheet (Alugram Xtra SIL G/UV254, Macherey-Nagel). Lipids were separated in a tank containing a solution of chloroform/methanol/acetic acid/water (50:30:8:2). Staining was performed with iodine vapor.

### Dot blotting

Particles were prepared as above, using DMEM (1 ml) containing 0.04–20 μg of xanthan gum. Particles were treated with EDTA, and the solution (20 μl) was transferred to polyvinylidene difluoride (PVDF) membranes using a dot blot apparatus (Bio-Rad). PVDF membranes were incubated into a blocking solution of phosphate buffered saline (PBS) containing 0.05% (v/v) Tween 20 and 3% (w/v) BSA at room temperature for 2 hrs, and probed with 15 ml of PBS containing 0.2 μg of biotinylated wheat germ agglutinin (WGA; Sigma) at room temperature for 1 hr. After two washing steps using 20 ml of blocking solution containing 1% (w/v) of BSA for 5 min, the PVDF membranes were incubated with 15 ml of blocking buffer containing streptavidin-horseradish peroxidase (HRP) antibody (ab7403, Abcam; 1:25,000) at room temperature with gentle agitation for 30 min. Membranes were washed four times with 20 ml of blocking solution. Signal was revealed with 1 ml of luminol enhancer solution (Promega) based on instructions provided by the manufacturer. Signal was revealed using X-ray radiographic films.

### Agarose gel electrophoresis

Particles were prepared as above, using DMEM (1 ml) containing 1–30 μg of salmon sperm DNA. Particles were treated with EDTA, and the sample (20 μl) was submitted to agarose gel electrophoresis. Gels were stained with ethidium bromide and photographed over a UV trans-illuminator using a digital camera (FluorChem Q System, ProteinSimple).

### Proteomic analysis

A washed particle specimen corresponding to each condition was resuspended in 50 mM EDTA and treated with a solution of 25 mM NH_4_HCO_3_ containing 10 mM dithiothreitol at 56 °C for 45 min. Samples were treated with 25 mM NH_4_HCO_3_ containing 55 mM iodoacetamide at 22 °C for 30 min. Proteins were digested with 20 μg/ml sequencing-grade porcine trypsin (Promega) at 37 °C overnight. The samples were desalted using a homemade desalting column, prior to drying in a vacuum centrifuge, and resuspension in 0.1% (w/v) formic acid. Samples were loaded into a reverse-phase liquid chromatography trap column (Agilent Technologies, Zorbax 300SB-C18). Peptide separation was performed using a 10-cm analytical C18 column (New Objective, 75 μm internal diameter). Elution was performed by gradually increasing the concentration of acetonitrile (ACN), starting with 2% (v/v) ACN in 0.1% formic acid and ending with 95% ACN. The column was coupled to a LTQ-Orbitrap mass spectrometer operated using the Xcalibur 2.0 software (Thermo Fisher Scientific). Proteomic analysis was performed as described earlier^7^.

### Metabolomic analysis

Ultra-performance liquid-chromatography (UPLC) was performed using an Acquity BEH C18 column (Waters; 130 Å, 1.7 μm, 2.1 mm × 100 mm). Mass spectrometry was done using time-of-flight (TOF) Synapt HDMS system (Waters). UPLC-grade solvents were purchased from Fluka. Capillary and cone voltage were set at 3000 V (2000 V in ESI^−^ mode) and 35 V, respectively. Desolvation gas flow rate was 700 l/h, and gas flow was maintained at 25 l/h. Desolvation and source temperatures were respectively set at 300 °C and 80 °C. LockSpray frequency was set at 0.5 sec and was averaged over 10 scans for correction. Metabolites were validated by comparing candidate MS spectra with that of metabolite standards (Sigma). Data analysis of a sample corresponding to each condition as well as compound identification were performed as before^17^.

## Results

**Figure 1:**
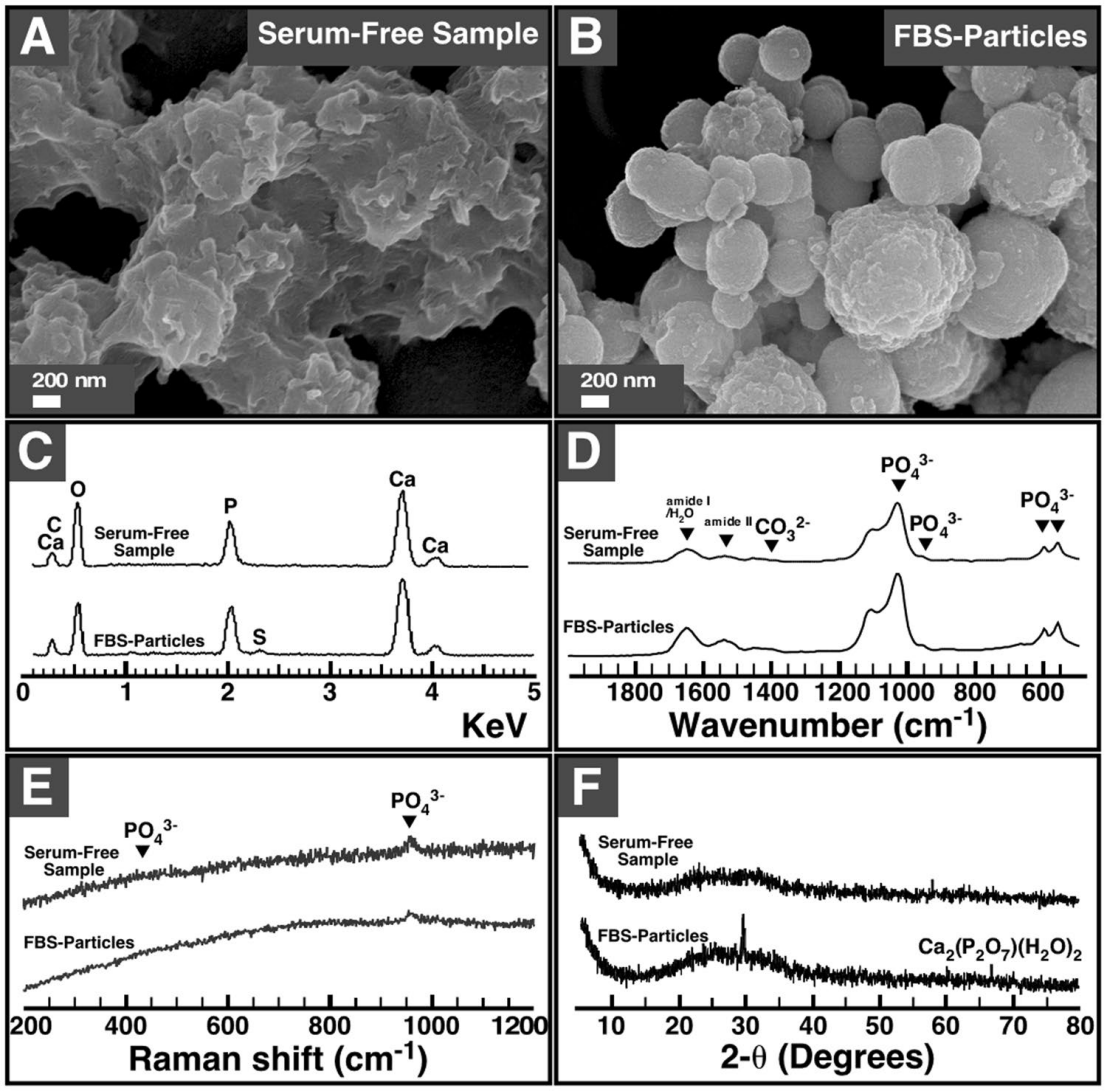
Morphology and chemical composition of mineralo-organic particles used in the present study. Specimens were prepared by adding 3 mM of CaCl_2_ and Na_2_HPO_4_ each into DMEM with or without 10% FBS as described in the Methods. Following incubation in cell culture conditions overnight, the specimens were prepared for (**A**,**B**) SEM, (**C**) EDX spectroscopy, (**D**) FTIR spectroscopy, (**E**) Raman spectroscopy, and (**F**) powder X-ray diffraction analysis. The images and spectra shown here and in the other figures are representative results of experiments performed in triplicate.

We observed earlier that mineral NPs prepared by mixing calcium and phosphate ions into a cell culture medium (i.e., DMEM) containing a body fluid such as serum are similar to the mineral particles detected in the human body in terms of their size, morphology, structure and composition^15^. We therefore used these mineral particles to determine the organic compounds that bind to biological particles in body fluids of animals and humans. We also examined specimens prepared without serum in order to assess the effects of the latter on particle formation. Under scanning electron microscopy (SEM), specimens prepared by adding calcium and phosphate into DMEM in the absence of serum produced mineralized biofilm-like structures in which no distinct particles were discernible (Fig. 1A, “Serum-Free Sample”). However, in the presence of FBS, the specimens contained round NPs and microparticles with diameters of 50 nm to 1 μm (Fig. 1B, “FBS-Particles”), consistent with our initial observations that organic compounds such as proteins induce the formation of round and amorphous mineral particles in biological fluids^2,3^.

We used spectroscopy analysis to examine the composition of the particles. Under EDX spectroscopy, the samples prepared in the presence or absence of FBS showed peaks of carbon, calcium, oxygen and phosphorus (Fig. 1C). A minor peak of sulfur was also noticed in FBS-particles (Fig. 1C). We used FTIR spectroscopy to determine the chemical functional groups found in the mineral phase of the prepared samples. Serum-free samples and FBS-particles showed peaks corresponding to water and amide bonds (possibly due to the presence of water, DMEM components, and/or serum proteins), in addition to carbonate and phosphate groups (Fig. 1D). Phosphate peaks were also detected in serum-free samples and FBS-particles using Raman spectroscopy (Fig. 1E). While no crystalline peaks were observed in the X-ray diffraction pattern of serum-free samples, FBS-particles showed a minor peak corresponding to calcium phosphate (Fig. 1F).

**Figure 2:**
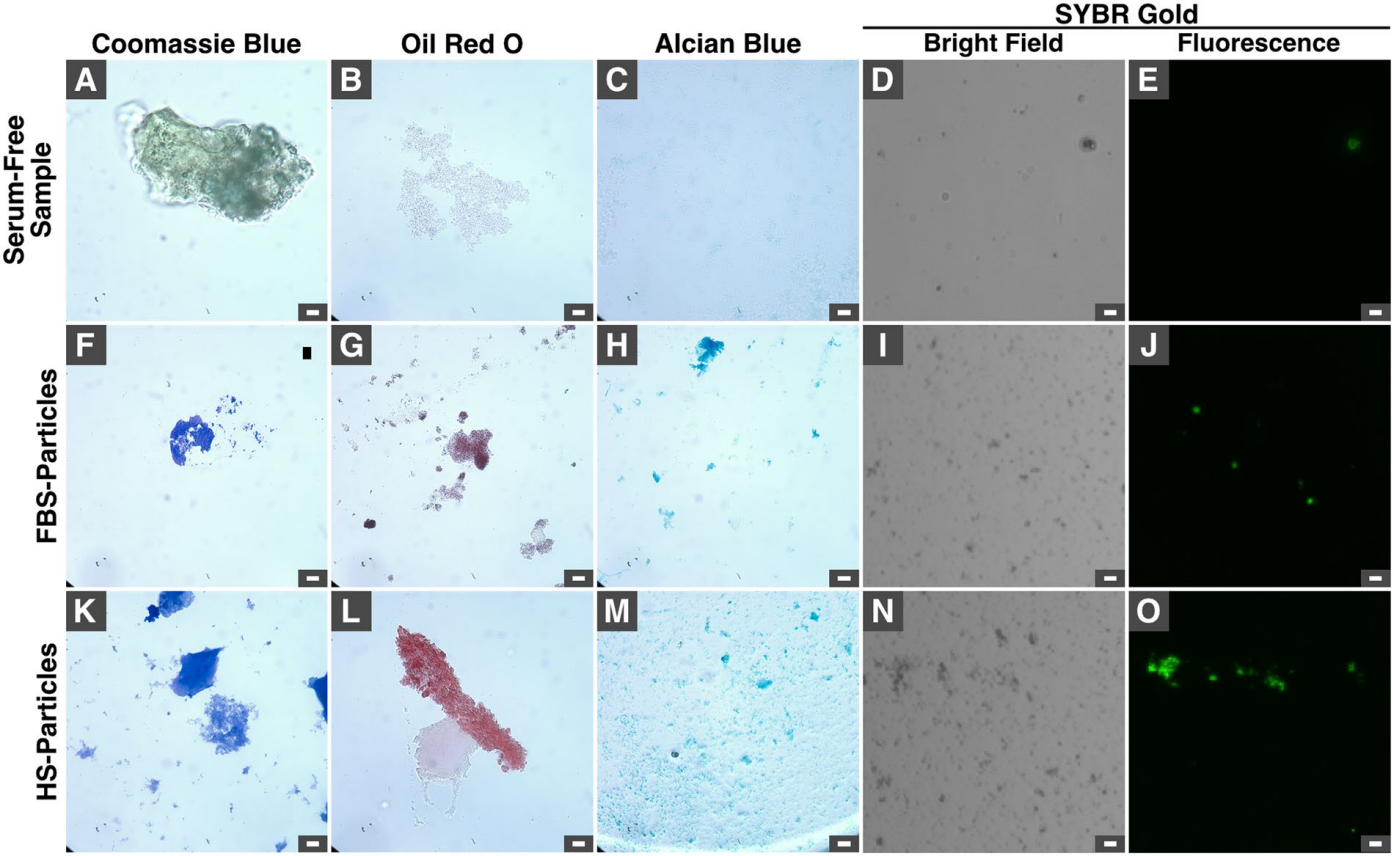
Staining of mineralo-organic particles observed under optical microscopy. Specimens were prepared by adding 3 mM of CaCl_2_ and Na_2_HPO_4_ each into DMEM with or without 10% FBS or HS, followed by washing and staining with dyes that bind to proteins (Coomassie blue), lipids (oil red O), polysaccharides (alcian blue) or DNA (SYBR gold) as described in Methods. Stained specimens were observed under an optical microscope with a bright field condenser (**A**–**D**,**F**–**I**,**K**–**N**) or with an epi-fluorescence microscope (**E**,**J**,**O**). Scale bars: 10 μm.

We used chemical dyes such as Coomassie blue, oil red O, alcian blue and SYBR gold in order to determine the presence of proteins, lipids, polysaccharides, and DNA, respectively, within the particles. While serum-free mineral samples did not stain with any of these dyes (Fig. 2A–E; minor positive staining was attributed to auto-fluorescence in E), particles prepared in the presence of FBS or HS (“HS-Particles”) showed positive staining for all chemical dyes tested (Fig. 2F–O). These results indicate that the main classes of organic compounds are present within the particles.

**Figure 3:**
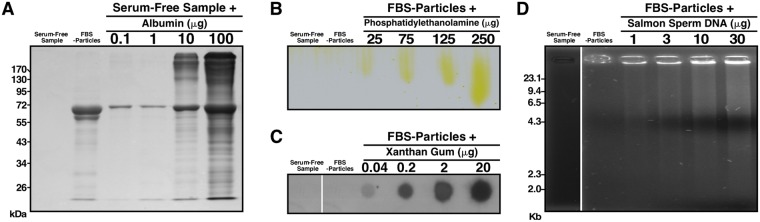
Mineral particles bind to various organic molecules. Specimens were prepared by adding 3 mM of CaCl_2_ and Na_2_HPO_4_ each into DMEM (1 ml, final volume) containing (**A**) bovine serum albumin, (**B**) phosphatidylethanolamine, (**C**) xanthan gum, or (**D**) salmon sperm DNA at the amounts indicated. The samples were washed in 20 mM HEPES buffer and resuspended in 50 μl of 50 mM EDTA. Specimens were prepared for (**A**) SDS-PAGE, (**B**) thin-layer chromatography, (**C**) dot blot and (**D**) agarose gel as described in Methods. Vertical white lines in (**C**,**D**) were used to separate blots or gels obtained in identical conditions. The full gels or blots are shown.

To confirm the binding of organic compounds to mineral particles, we prepared specimens in cell culture medium containing purified organic compounds. While serum-free specimens did not show any protein staining as assessed by SDS-PAGE, FBS-particles stained for proteins (Fig. 3A). Similarly, serum-free specimens prepared in the presence of albumin showed dose-dependent protein staining (Fig. 3A). Serum-free specimens and FBS-particles did not show the presence of the phospholipid phosphatidylethanolamine (PE) as assessed by thin-layer chromatography; however, FBS-particles prepared in the presence of PE showed dose-dependent PE staining (Fig. 3B). We also prepared mineral samples in the presence of the polysaccharide xanthan gum, and performed dot blots with biotinylated wheat germ agglutinin to determine the binding of the polysaccharide to the particles (Fig. 3C). While serum-free specimens and FBS-particles showed no detectable staining, FBS-particles prepared in the presence of xanthan gum showed dose-dependent staining for the polysaccharide (Fig. 3C). Experiments were also performed to verify whether DNA bind to the mineral particles. Agarose gel electrophoresis showed that, as expected, serum-free samples prepared in the absence of DNA were not stained by ethidium bromide; however, FBS-particles and FBS-particles prepared in the presence of DNA showed positive staining (Fig. 3D). These results indicate that mineralo-organic particles bind to proteins, phospholipids, polysaccharides and DNA.

In order to determine the organic molecules that bind to the particles in a comprehensive manner, we submitted the particles to proteomics analysis. Following incubation and washing steps, the particles were resuspended in EDTA to release proteins. Various proteins were shown to bind to the mineral particles, including calcification inhibitors (i.e., fetuin-A, albumin), complement proteins (i.e., C3, C4-A), coagulation factors (i.e., factor X, prothrombin), glycoproteins (i.e., histidine-rich glycoprotein, vitronectin), apolipoproteins (i.e., A1, B-100, clusterin), and protease inhibitors (i.e., inter-α-trypsin inhibitor) (Table 1). While the proteins that bind to HS-particles and FBS-particles were similar, HS-particles harbored more proteins than FBS-particles (Table 1), possibly due at least in part to the higher concentration of proteins in HS (60 mg/ml) compared with FBS (32 mg/ml), as noted earlier^4^.

**Table 1:** Protein composition of serum-derived particles.

No.	Protein	Accession No.	MW (kDa)	HS-Particles		FBS-Particles	
				emPAI	Spectra	emPAI	Spectra
1	Fetuin-A	P12763	38.39	—	—	1.1	103.9
2	Complement C3	P01024	187.03	0.17	21	—	—
3	Albumin	P02768	69.32	0.19	19.9	—	—
4	Fetuin-A	P02765	39.30	0.37	15.5	—	—
5	Vitronectin	P04004	54.27	0.17	10	—	—
6	Inter-α-trypsin inhibitor heavy chain H2	P19823	106.40	0.17	8.9	—	—
7	Apolipoprotein B-100	P04114	515.28	0.02	8.9	—	—
8	Albumin	P02769	69.25	—	—	0.35	8.2
9	Prothrombin	P00734	69.99	0.1	6.6	—	—
10	Complement C4-A	P0C0L4	192.66	0.04	5.5	—	—
11	Coagulation factor X	P00743	54.48	—	—	0.3	4.6
12	Coagulation factor X	P00742	54.70	0.2	4.4	—	—
13	Clusterin	P10909	52.46	0.17	4.4	—	—
14	Apolipoprotein A1	P02647	30.76	0.09	4.4	—	—
15	Histidine-rich glycoprotein	P04196	59.54	0.07	4.4	—	—
16	C4b-binding protein α chain	P04003	66.99	0.06	4.4	—	—
17	Serum amyloid A-4 protein	P35542	14.74	0.74	3.3	—	—
18	Prothrombin	P00735	70.46	—	—	0.12	3.6
19	Prothymosin-α	P01252	12.06	—	—	1	2.7
20	Complement C3	Q2UVX4	187.14	—	—	0.07	3.6
21	α-1-Antitrypsin	P01009	46.71	0.09	3.3	—	—
22	Afamin	P43652	69.02	0.06	3.3	—	—
23	Serotransferrin	Q29443	77.70	—	—	0.11	1.8

Proteins were ranked based on the sum of emPAI and spectral counts (“spectra”) for HS-particles and FBS-particles. Accession numbers correspond to UniProt entries. MW, molecular weight; emPAI, exponentially modified protein abundance index.

We also performed a metabolomics analysis in order to identify the low-molecular weight compounds that bind to the mineral particles. Organic compounds released from the particles were resuspended in methanol and analyzed using reversed-phase UPLC-MS analysis. In the ESI^+^ mode, the compounds found to bind to the mineral particles included lyso-phosphatidylcholines (lysoPCs), monoacyl-glycerols (MGs), amides (i.e., lauroyldiethanolamide, stearoyldiethanolamide, niacinamide), carbohydrates (i.e., glucose) and amino acids (i.e., L-leucine, L-phenylalanine) (Table 2). In the ESI^−^ mode, the organic compounds identified included mainly lysoPCs and fatty acids (i.e., stearic acid, palmitic acid, oleic acid, myristic acid, margaric acid, pentadecylic acid, palmitoleic acid, linolenic acid, 9-heptadecenoic acid, arachidic acid, docosahexaenoic acid) (Table 3). While HS-particles and FBS-particles interacted with the same organic compounds, variation in signal intensity was noted between these samples (Tables 2 and 3).

**Table 2:** Organic compounds bound to serum-derived particles and identified in the ESI^+^ mode.

#	Compound	ID Number	Formula	RT (min)	m/z	FBS-Particles	HS-Particles
1	LysoPC (16:0)	HMDB10382	C_24_H_50_NO_7_P	2.09	496.3402	3639.3	18967.7
2	MG (18:0)	HMDB11535	C_21_H_42_O_4_	2.57	359.3141	1389.6	1624.1
3	MG (16:0)	HMDB11564	C_19_H_38_O_4_	2.41	331.2822	1096.1	815.7
4	LysoPC (20:4)	HMDB10395	C_28_H_50_NO_7_P	1.94	544.3405	733.9	5835.1
5	LysoPC (18:1)	HMDB02815	C_26_H_52_NO_7_P	2.10	522.3574	442.4	1019.3
6	Glucose	HMDB06564	C_6_H_11_O_6_Na	0.48	203.0523	312.6	645.6
7	LysoPC (22:6)	HMDB10404	C_30_H_50_NO_7_P	1.91	568.3402	251.9	2374.4
8	Lauroyldiethanolamide	HMDB32358	C_16_H_33_NO_3_	1.84	288.2536	122.2	140.6
9	Stearoylethanolamide	HMDB13078	C_20_H_41_NO_2_	2.53	328.3184	105.9	101.2
10	Choline	HMDB00097	C_5_H_14_NO	0.52	104.1069	78.9	434.2
11	Niacinamide	HMDB01406	C_6_H_6_N_2_O	0.84	123.0564	54.9	139.1
12	L-Phenylalanine	HMDB00159	C_9_H_11_NO_2_	0.85	166.0867	47.7	154.7
13	L-Leucine	HMDB00687	C_6_H_13_NO_2_	0.81	132.1022	38.6	65.2

Numbers given within parentheses in the “Compound” column correspond respectively to the number of carbons and double bonds found in the fatty acid molecule. Compounds were ranked based on the ion intensity of FBS-particles. ESI, electrospray ionization; HMDB, Human Metabolome Database; ID, identification; MG, mono-acylglycerol; PC, phosphatidylcholine; RT, retention time.

**Table 3:** Organic compounds bound to serum-derived particles and identified in the ESI^−^ mode.

#	Compound	ID Number	Formula	RT	m/z	FBS-Particles	HS-Particles
1	Stearic acid	HMDB00827	C_18_H_36_O_2_	2.80	283.2628	15500.5	16917.9
2	Palmitic acid	HMDB00220	C_16_H_32_O_2_	2.50	255.2317	11803.8	14896.4
3	Oleic acid	HMDB00207	C_18_H_34_O_2_	2.51	281.2487	1593.3	4197.2
4	Myristic acid	HMDB00806	C_14_H_28_O_2_	2.27	227.2003	1500.3	411.6
5	Margaric acid	HMDB02259	C_17_H_34_O_2_	2.66	269.2479	1355.6	1698.2
6	Pentadecylic acid	HMDB00826	C_15_H_30_O_2_	2.39	241.2156	1127.3	711.7
7	Palmitoleic acid	HMDB03229	C_16_H_30_O_2_	2.30	253.2159	624.6	548.1
8	Linolenic acid	HMDB00673	C_18_H_32_O_2_	2.33	279.2325	331.4	6627.5
9	9-Heptadecenoic acid	HMDB31046	C_17_H_32_O_2_	2.40	267.2325	207.1	155.7
10	Arachidic acid	HMDB02212	C_20_H_40_O_2_	3.31	311.2954	206.8	179.6
11	LysoPC (15:0)	HMDB10381	C_23_H_48_NO_7_P	2.07	480.3085	199.1	839.4
12	LysoPC (18:2)	HMDB10386	C_21_H_48_N_11_O_3_PS	1.99	564.3322	101.5	1871.3
13	DHA	HMDB61088	C_22_H_32_O_2_	2.21	327.2322	77.2	1558.5

Numbers given within parentheses in the “Compound” column correspond respectively to the number of carbons and double bonds found in the fatty acid molecule. Compounds were ranked based on the ion intensity of FBS-particles. ESI, electrospray ionization; HMDB, Human Metabolome Database; ID, identification; DHA, docosahexaenoic acid; PC, phosphatidylcholine; RT, retention time.

## Discussion

Major advances have been made in the understanding of the protein corona which may influence the effects of NPs in the human body^22,23^. On the other hand, it remained unclear whether other organic molecules may also bind to mineral NPs that form in body fluids. We describe here that the major classes of organic molecules, including proteins, sugars, lipids, polysaccharides and nucleic acids, bind to mineralo-organic particles derived from biological fluids. These organic molecules are likely to stabilize the formation of mineral particles and allow their transport in biological fluids, in a manner similar to what has been described earlier regarding the role of proteins in particle formation^5,13^. While earlier studies have shown that the particles prepared *in vitro* using the approach described in the present study are highly similar to the mineralo-organic particles detected in human tissues^2–4,15^, we could not exclude the possibility that minor differences may be observed between the organic molecules that bind to the prepared and isolated particles. Our results suggest that the binding of these organic compounds to particles may influence various physiological and pathological processes occurring *in vivo*, a possibility that remains to be examined in more details.

One of the major findings reported here is that mineralo-organic particles prepared in biological fluids bind to nucleic acids. These mineral-DNA complexes show similarities to virus particles in terms of their sizes and chemical composition. Mineral particles consisting of calcium phosphate are routinelly used for transfection of DNA into eukaryotic cells in culture^24^. Calcium phosphate particles have also been used as gene-delivery vectors *in vivo*^25^. These observations suggest that cells may internalize mineralo-organic particles in the circulation and in tissues, possibly leading to transfer of DNA between host cells and other organisms, including the gut microbiota. It remains to be seen whether this phenomenon occurs *in vivo* and whether it may produce any effects on the cells and organs in the body.

We propose that the organic compounds found to bind to the mineralo-organic particles may serve as markers to detect and quantify the particles in human tissues and body fluids, as well as to study the formation and role of the biological particles in the human body. Accordingly, proteins such as albumin and fetuin-A have been shown to consistently interact with the mineral particles that form in biological fluids^2,4,7,13^. Spectroscopy analyses such as EDX and X-ray diffraction may thus be used in combination with enzyme-linked immunosorbent assay (ELISA), proteomics or metabolomics to detect mineralo-organic particles in biological specimens. Moreover, we expect that the methodology described here will also yield important information regarding the effects of mineralo-organic particles in the body.

## Conclusion

Our results indicate that mineralo-organic particles that form in biological fluids bind to various organic compounds. The major families of organic molecules were all found to bind to the mineral particles, including proteins, lipids, polysaccharides and DNA. The profiling performed in the present study provides a platform to study the effects of such molecule-particle interactions in physiological and pathological processes occurring in the human body.
